# An optimised age-based dosing regimen for single low-dose primaquine for blocking malaria transmission in Cambodia

**DOI:** 10.1186/s12916-016-0701-8

**Published:** 2016-10-27

**Authors:** Rithea Leang, Naw Htee Khu, Mavuto Mukaka, Mark Debackere, Rupam Tripura, Soy Ty Kheang, Say Chy, Neeraj Kak, Philippe Buchy, Arnaud Tarantola, Didier Menard, Arantxa Roca-Felterer, Rick M. Fairhurst, Sim Kheng, Sinoun Muth, Song Ngak, Arjen M. Dondorp, Nicholas J. White, Walter Robert John Taylor

**Affiliations:** 1 National Center for Parasitology, Entomology and Malaria Control, Corner St. 92, Trapeng Svay Village, Sangkat Phnom Penh, Thmei, Khan Sen Sok, Phnom Penh, Cambodia; 2 Mahidol Oxford Tropical Medicine Research Unit (MORU), 420/6 Rajvithi Road, Rajthevee, Bangkok, 10400 Thailand; 3 Oxford Centre for Tropical Medicine and Global Health, Nuffield Department of Medicine Research Building, University of Oxford, Old Road Campus, Roosevelt Drive, Oxford, OX3 7FZ UK; 4 MSF Belgium Cambodia Malaria Program, #19, Street 388, Sangkat Tuol Svay Prey, Khan Chamkarmon, PO Box 1933, Phnom Penh, Cambodia; 5 University Research Co., LLC, MK Building, House #10 (2nd floor), St. 214, Chey Chumneas, Daun Penh, Phnom Penh, Cambodia; 6 University Research Co., LLC Washington DC: 7200 Wisconsin Ave, Bethesda, MD 20814 USA; 7 Institut Pasteur du Cambodge, 5 Monivong Boulevard, PO Box 983, Phnom Penh, 12201 Cambodia; 8 Malaria Consortium, House #91 Street 95, Boeung Trabek, Chamkar Morn, Phnom Penh, Cambodia; 9 Laboratory of Malaria and Vector Research, National Institute of Allergy and Infectious Diseases, National Institutes of Health, Rockville, MD 20852 USA; 10 FHI 360 Cambodia Office, #03, Street 330 Boeung Keng Kang III Khan Chamkamon, PO Box: 2586, Phnom Penh, Cambodia; 11 Centre de Médecine Humanitaire, Hôpitaux Universitaires de Genève, Genève, Switzerland

**Keywords:** Primaquine, Malaria, G6PD deficiency, Dosing, Cambodia

## Abstract

**Background:**

In 2012, the World Health Organization recommended the addition of single low-dose primaquine (SLDPQ, 0.25 mg base/kg body weight) to artemisinin combination therapies to block the transmission of *Plasmodium falciparum* without testing for glucose-6-phosphate dehydrogenase deficiency. The targeted group was non-pregnant patients aged ≥ 1 year (later changed to ≥ 6 months) with acute uncomplicated falciparum malaria, primarily in countries with artemisinin-resistant *P. falciparum* (ARPf). No dosing regimen was suggested, leaving malaria control programmes and clinicians in limbo. Therefore, we designed a user-friendly, age-based SLDPQ regimen for Cambodia, the country most affected by ARPf.

**Methods:**

By reviewing primaquine’s pharmacology, we defined a therapeutic dose range of 0.15–0.38 mg base/kg (9–22.5 mg in a 60-kg adult) for a therapeutic index of 2.5. Primaquine doses (1–20 mg) were tested using a modelled, anthropometric database of 28,138 Cambodian individuals (22,772 healthy, 4119 with malaria and 1247 with other infections); age distributions were: 0.5–4 years (20.0 %, n = 5640), 5–12 years (9.1 %, n = 2559), 13–17 years (9.1 %, n = 2550), and ≥ 18 years (61.8 %, n = 17,389). Optimal age-dosing groups were selected according to calculated mg base/kg doses and proportions of individuals receiving a therapeutic dose.

**Results:**

Four age-dosing bands were defined: (1) 0.5–4 years, (2) 5–9 years, (3) 10–14 years, and (4) ≥15 years to receive 2.5, 5, 7.5, and 15 mg of primaquine base, resulting in therapeutic doses in 97.4 % (5494/5640), 90.5 % (1511/1669), 97.7 % (1473/1508), and 95.7 % (18,489/19,321) of individuals, respectively. Corresponding median (1st–99th centiles) mg base/kg doses of primaquine were (1) 0.23 (0.15–0.38), (2) 0.29 (0.18–0.45), (3) 0.27 (0.15–0.39), and (4) 0.29 (0.20–0.42).

**Conclusions:**

This age-based SLDPQ regimen could contribute substantially to malaria elimination and requires urgent evaluation in Cambodia and other countries with similar anthropometric characteristics. It guides primaquine manufacturers on suitable tablet strengths and doses for paediatric-friendly formulations. Development of similar age-based dosing recommendations for Africa is needed.

**Electronic supplementary material:**

The online version of this article (doi:10.1186/s12916-016-0701-8) contains supplementary material, which is available to authorized users.

## Background

In September 2012, the World Health Organization (WHO) Evidence Review Group recommended adding single low-dose primaquine (SLDPQ, 0.25 mg base/kg body weight, 15 mg for adults) to artemisinin combination therapies (ACTs), without testing for glucose-6-phosphate dehydrogenase deficiency (G6PDd), in non-pregnant, falciparum-infected patients aged ≥ 1 year (later amended to ≥ 6 months) to block *Plasmodium falciparum* gametocyte transmission from humans to mosquitoes [1, 2]. This recommendation was aimed primarily at countries of the Greater Mekong Subregion (GMS) where artemisinin-resistant *P. falciparum* (ARPf) is well established and continues to emerge [3–9]. Indeed, ARPf is now at the threshold of the Indian subcontinent [10].

The 2012 recommendation was pragmatic, taking into account the lack of detailed, carefully conducted dose-optimisation studies of primaquine (PQ), the reluctance to use PQ in the GMS because of the fear of PQ-induced acute haemolytic anaemia (AHA) in G6PDd patients, the logistical impossibility of testing widely for G6PDd, and the urgency to eliminate ARPf in the GMS. There is now convincing evidence that increasing treatment failures of dihydroartemisinin-piperaquine (DHAPP), the first-line ACT in Cambodia, is due to a combination of ARPf and PP resistance and that ARPf has probably contributed to the rapidly evolving ineffectiveness of this ACT [11–14]. More ACT failures can be expected in areas of ARPf and this alarming scenario underscores the urgency to eliminate falciparum malaria from the GMS.

Aside from Vietnam, which uses 0.75 mg base/kg of PQ, countries in the GMS have been slow to recommend SLDPQ, but attitudes are changing. In August 2015, Cambodia decided to deploy SLDPQ thanks, in part, to reassuring data on the use of 0.75 mg base/kg of weekly PQ (45 mg in a 60-kg adult) in vivax-infected G6PDd patients [15]. Moreover, a pilot study of SLDPQ is being conducted in acute uncomplicated falciparum malaria (ClinicalTrials.gov identifier, NCT02434952) and SLDPQ is being included in antimalarial drug resistance studies (NCT02453308).

When countries agree to adopt SLDPQ, one fundamental question is how the recommended dose is translated into a practical dosing regimen. No PQ dosing regimen was suggested in the 2012 recommendation. The 2015 WHO Malaria Treatment Guidelines (http://www.who.int/malaria/publications/atoz/9789241549127/en/) contain a weight-based regimen using whole and fractions of a 7.5-mg PQ base tablet; it is unclear what evidence informed this regimen. Weight-based regimens that require tablet fractions are a major hassle for care providers and result in poor dosing accuracy [16].

In Cambodia, approximately one-third of malaria patients receive treatment through Cambodia’s network of village malaria workers, who do not have weighing scales (D. Soley, personal communication), thus limiting the impact of weight-based regimens. Moreover, Cambodian patients frequently buy drugs from market stalls and village drug shops [17, 18], a common practice seen in other malaria-endemic countries [19, 20]. The PQ dosing conundrum is not new and one solution is to design easy to use, optimised, age-based dosing regimens, as has been done for artesunate-amodiaquine [21], artesunate-mefloquine, and DHAPP.

Designing such regimens is not straightforward. Many elements must be considered carefully, including drug pharmacokinetics (PK) and pharmacodynamics (PD, i.e. efficacy and toxicity), PK–PD relationships, simplicity of use, and anthropometric differences between countries [21, 22]. Ultimately, the crucial determination is the mg base/kg dosing therapeutic range. Dosing within the range is considered optimal but underdosing or supratherapeutic doses risk low efficacy and dose-dependent drug toxicity, respectively. Drugs with lower therapeutic indices offer less dosing flexibility. Herein, we propose an age-based SLDPQ regimen for Cambodia and report on how we arrived at this regimen.

### The efficacy of low-dose PQ in reducing mosquito infectivity

Since the 1920s, pioneering work in falciparum malaria patients showed that low doses of the 8-aminioquinoline plasmochin/e (also known as pamaquine/plasmoquine) rapidly reduced mosquito infectivity (typically ≤ 24 h), well before the 3–4 days it took to clear gametocytes [23, 24]. Moreover, recent data from Mali has shown no relationship between gametocyte density and infectivity after PQ administration [25]. Thus, gametocyte parameters should be considered weak surrogate markers of mosquito infectivity [26].

**Fig. 1:**
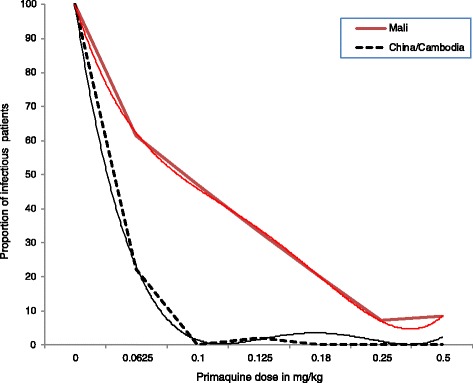
Mosquito infectivity as a function of administered primaquine dose. The trends have been smoothened using polynomials of order 4 and 6 for Mali and China/Cambodia, respectively. The red line represents Malian patients treated with dihydroartemisinin-piperaquine (DHAPP) [25], and the green line represents Vietnamese and Cambodian adults treated with DHAPP [1]

In the 1960s, limited data in naturally- [27] and experimentally-acquired [28] falciparum malaria showed that 15 mg of PQ (i.e. SLDPQ) reduced mosquito infectivity to a similar degree as 45 mg of PQ, but the latter became the WHO-recommended dose. Data from *P. falciparum*-infected, DHAPP-treated, Vietnamese and Cambodian adults show a curvilinear response between PQ dose (3.7–15 mg base) and mosquito infectivity, assessed by mosquito membrane feeding assays [1]; 7.5 mg of PQ base (0.125 mg base/kg in a 60-kg individual) provided almost maximal transmission blocking, suggesting this dose is close to the minimum dose that produced the maximum effect – the minimum transmission-blocking (MTB) dose. Support that 0.125 mg base/kg is close to the MTB comes from mosquito membrane feeding assays in DHAPP-treated, *P. falciparum*-infected Malians (Fig. 1), in whom the MTB was between 0.125 and 0.25 mg base/kg; 0.0625 mg base/kg was similar to no PQ [25].

Data from mass drug administration (MDA) in 17 Cambodian villages using an initial dose of artemisinin-piperaquine plus 9 mg of PQ (0.15 mg base/kg in a 60 kg person) followed by PQ administered every 10 days for 6 months reduced malaria prevalence rates over 3 years [29]. Although lacking a control group, mathematical modelling supported the reduction in *P. falciparum* transmission with this PQ regimen [30]. Based on these efficacy data, the MTB dose of PQ lies between 0.125 and 0.15 mg/kg of PQ base.

### Primaquine tolerability and safety

An extensive review of PQ’s safety has shown that it is very well tolerated. Deaths are extremely rare and associated with repeated PQ doses [31]. However, there are few data on SLDPQ and very limited data on PQ in South East Asian variants of G6PDd.

G6PDd, an X-linked inherited red blood cell enzymopathy affecting 400 million people, is a major public health challenge to malaria elimination because PQ-induced AHA is dose-dependent and related to the degree of enzyme deficiency [32, 33]. Therefore, hemizygous males (X*Y) and homozygous females (X*X*) generally suffer greater AHA compared to heterozygous females (X*X), and AHA is milder in the African A- variant compared to more severe G6PDd variants such as Mediterranean, Viangchan, Mahidol, Canton, and Hong Kong-Pokfulam [34–38]. Recognising the paucity of safety data, the WHO Evidence Review Group relied on expert opinion, stating “*we expect that a single 15 mg PQ adult dose (0.25 mg base/kg) will not result in clinically-significant haemolysis in G6PD-deficient individuals*” http://www.who.int/malaria/mpac/sep2012/primaquine_single_dose_pf_erg_meeting_report_aug2012.pdf?ua=1.

In Cambodia, approximately 90 % of G6PDd is due to the Viangchan variant, which has a median enzyme activity of 0.8 U/gHb (~7 % of the population median of 12 U/gHb), placing it at the severe end of the G6PDd spectrum [39, 40]. AHA induced by 15 mg of daily PQ alone was well tolerated in healthy Cambodian Air Force men who had a mean baseline haemoglobin concentration of approximately 14 g/dL and which fell by a mean of 21 % on day (D)7, persisting to D15 [37]. Data in vivax-infected patients, with Hb concentrations ≥ 10 g/dL, showed that weekly-administered 0.75 mg base/kg of PQ resulted in a greater Hb level decline in G6PDd compared to G6PD normal patients. The greatest fractional fall in Hb occurred on D7 (median (range)): –16.3 % (–33.1 to 6.5) versus –3.7 % (–17.5 to 23.3) for corresponding absolute Hb concentration falls of –2.2 g/dL (–4.9 to 0.8) versus –0.5 g/dL (–2.2 to 2.8) [15].

The PQ dose-AHA response curve in G6PDd individuals is unknown, but the more severe variants will have a steeper slope because they are more sensitive to PQ’s oxidative effects [32]. Extrapolating the Hb data from the vivax-infected Cambodians and assuming similar Hb dynamics in patients with falciparum and vivax malaria [41], 0.38 mg/kg of PQ base (i.e. approximately half the dose given to the vivax malaria patients) might result in a fractional decline of up to approximately –20 % (approximately –2.5 g/dL). This decline should be well tolerated and is consistent with Hb declines seen in falciparum malaria patients in South East Asia, where approximately 60 % of ACT-treated falciparum malaria patients (with unknown G6PD status) have fractional declines in Hb of < 0 to –20 %, and 12 % have a decline exceeding –20 % (W. Taylor, unpublished observations).

However, approximately 5 and 12 % of Cambodian patients with acute uncomplicated falciparum or vivax malaria have Hb concentrations < 5 and < 7 g/dL, respectively (analysis from [41]); most are treated as outpatients with oral ACTs. The effect of SLDPQ in such patients with G6PDd remains unknown and demands caution in setting the upper end of the therapeutic dose range.

MDA data from villages on the Thai-Burmese border show that G6PDd and G6PD normal, healthy individuals had small declines in mean Hb of approximately –5 % and –1 %, respectively, when given DHAPP plus SLDPQ, the latter dosed exactly at 0.25 mg base/kg [42]. These data are reassuring for MDA but are not generalisable to malaria patients.

Methaemoglobinaemia (metHb, upper limit of normal approximately 2–3 %) is a dose-dependent side effect of PQ but is not considered a clinical worry because, in vivax infected patients, 0.75 mg base/kg of PQ resulted in a maximum metHb concentration of 4.9 %, a concentration that was well tolerated [15]; thus, 0.25 mg/kg of PQ is likely to result in very small increases in metHb. Abdominal pain, also dose-related, is reduced by food intake [43, 44] and was not an important symptom when 30 mg of PQ was given daily with a small snack for 1 year in Indonesian adults [45].

### PQ pharmacokinetics

PQ is readily absorbed from the gastrointestinal tract [46, 47] and undergoes hepatic oxidative deamination to its principal, inactive metabolite, carboxyprimaquine (cPQ), chiefly by monoamine oxidase (MAO) A [48, 49]. Cytochrome (CYP) P450 isoenzymes 2D6, 2C19, and 3A4 are quantitatively less important than MAO A, but CYP 2D6 is fundamental to the PD effects of PQ because this pathway produces oxidative metabolites (OMs) that are responsible for AHA [50–54], dose-dependent oxidation of Hb [55, 56], PQ's antirelapse efficacy [48, 57] and, probably, its gametocytocidal effect.

OM concentrations are very low, unstable, and difficult to measure; thus, their PK profiles and their PK–PD relationships remain undocumented [48]. This is a considerable drawback because inferences about the PK characteristics of OMs are based on the PK characteristics of PQ and cPQ, neither of which is pharmacologically important for transmission blocking [58, 59].

CYP 450 2D6 activity is under polymorphic genetic control resulting in several metabolic patterns: poor (poor efficacy, less dose-related toxicity), intermediate, extensive (normal), and ultrarapid/ultraextensive (potentially enhanced efficacy and dose-related toxicity) [60]. The genetic bases for the metabolic phenotypes are complex and their clinical predictive value unclear [61, 62].

The population prevalence rates of the CYP 2D6 polymorphisms are unknown in Cambodia, but data from 125 *P. falciparum*-infected Cambodians found allele frequencies for CYP 2D6*4 (poor activity/metabolisers), 2D6*10 (intermediate), and 2D6*17 (intermediate) of 0, approximately 63 % and 4 %, respectively [63]. Although these data are in broad agreement with other small studies [64, 65], predicting the PD effects of SLDPQ at the population level remains problematic.

PQ and cPQ exposures, measured as the area under the concentration time curve, are linearly dose-related [47, 66]. The inter-individual variation in PQ and cPQ exposure generally ranges from approximately 1.75–4 [46, 67–69]. One study in healthy G6PD normal children aged 5–12 years from Papua New Guinea found PQ–PK characteristics equivalent to those in adults [66]. This is a potentially significant finding because a failure to appreciate that children have higher clearance rates of some antimalarial drugs compared to adults has resulted in under dosing of sulfadoxine-pyrimethamine and DHAPP [70, 71]. The Papua New Guinea data support the use of the same mg base/kg dose range for adults and children as young as 5 years and should result in comparable OM exposures.

PQ–PK parameters are similar between healthy volunteers and vivax malaria patients, Thais and Caucasians, and are unaffected by G6PD status [46, 47, 72–74]; data on sex differences are inconsistent [46, 74, 75]. PQ and cPQ exposures are increased by falciparum malaria (reduced PQ clearance) [69], food (26 % when given with bread and butter (28 g of fat)) [46], and chloroquine (PQ 24 %, cPQ 14 %) and DHAPP (PQ 25 %, cPQ 9 %), which could have competed for CYP 3A4, thereby diverting more PQ to the MAO A and, presumably, 2D6 pathways [67, 68]. There is no PK interaction between PQ and either artesunate-pyronaridine [76] or mefloquine [69, 77]; no PK interaction data exist for PQ and artemether-lumefantrine (AL).

Extrapolating from the above, OM PK characteristics will probably depend mostly on PQ dose and absorption and CYP 2D6 metabolic status; thus, selecting the lower mg/kg dose of PQ base should ensure that the majority of patients will achieve adequate OM concentrations to reduce infectivity.

### Artemisinin-resistant *Plasmodium falciparum* malaria

ARPf results in slower killing of asexual forms, leading to increased gametocytogenesis. Falciparum malaria patients from areas of ARPf in western Cambodia (Pailin and Pursat) had a higher risk (*P* < 0.01) of gametocytaemia at presentation (18.6 %) and on D7 (16.3 %) compared to patients from either northern (Preah Vihear) or eastern (Ratanakiri) Cambodia (5.4 and 4.8 %, respectively) [8].

However, at baseline, gametocytaemia distributions (median (interquartile range)) in patients with patent gametocytaemia were not significantly different (*P* = 0.44) between western (32 (16–176) /μL) and eastern/northern (64 (32–144) /μL) Cambodia (E. Ashley, unpublished observations). Therefore, it seems likely that, for now, SLDPQ should be equally effective at killing gametocytes and reducing infectivity independent of ARPf, recognising the limitations of gametocytaemia for predicting infectivity.

### Defining the PQ therapeutic dose range

The recommended target dose of PQ is 0.25 mg base/kg given once with an ACT. In practice, exact dosing is not possible and some patients will receive mg base/kg doses above or below the target dose. The questions to address when setting a therapeutic limit are (1) how many patients are likely to receive an ineffective PQ dose, and (2) how many patients would experience symptomatic AHA?

Given the MTB dose derived from the mosquito infectivity data, the field experience of low-dose PQ, the caution against using too low a dose and the possibility of poor PQ metabolism, we propose the lower end of the therapeutic range to be 0.15 mg base/kg (9 mg in a 60-kg adult). Considering the Hb data in the *P. vivax*-infected G6PDd patients, we propose an upper dose of 0.38 mg base/kg (22.5 mg in a 60-kg adult). These limits translate into a therapeutic index of 2.5.

## Methods

### Assembling the Cambodian anthropometric database

After obtaining permission from the Demographic Health Survey (DHS), the 2014 DHS Cambodian database was downloaded from the DHS web site (http://www.measuredhs.com). The database contains a broad range of data collected randomly. The data of prime interest were available in children aged < 60 months and women aged 15–45 years: (1) age (years and months for children < 60 months), (2) weight to one decimal place, (3) sex, (4) pregnancy status, (5) residential address, and (6) whether the residence was rural or urban.

Other databases, obtained from colleagues at the Centre National de Malariologie and several of its collaborators, consisted of malaria patients, field surveys of individuals in malaria-endemic villages, patients with suspected central nervous system (CNS) infections, and individuals with dog bites who presented to l’Institut Pasteur du Cambodge Rabies Prevention Centre in Phnom Penh. Colleagues and collaborators were first asked by WRJT if they would be willing to send relevant, anonymised anthropometric, demographic, and geographical data. They were informed why these data were being sought and that those who freely gave data would be co-authors on this paper.

Obtaining ethical clearance was not generally considered relevant for this study because most data (DHS) were already in the public domain, the dog bite data were routine clinic data, and the rest of the data were from studies approved by the National Ethical Committee for Health Research of the Cambodian Ministry of Health. In some studies, patients provided consent for secondary analyses so long as they were related to malaria. The Oxford Tropical Research Ethics Committee confirmed that this study was exempt from ethical review.

### Modelling the weight-for-age data

**Fig. 2:**
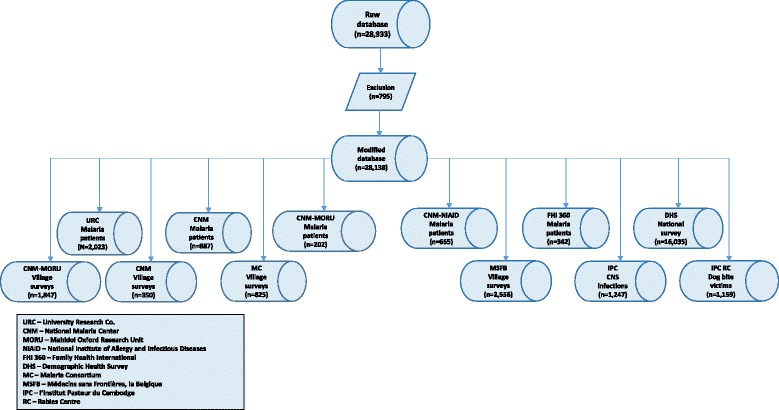
Database structure

The raw weight-for-age data were modelled into a growth curve (Additional file 1: Figure S1) by firstly excluding outlying weights for age; these were defined as values falling outside of the 1st and 99th centiles, using Box Cox power exponential model centiles. This resulted in 795 individuals being excluded from the database (Fig. 2). In order to obtain the weight-for-age centiles, the data were modelled using the three-parameter Box Cox power exponential distribution [78] and the centile growth curves smoothed using the cubic spline smoothing technique [79]. The modelling was performed in Stata using the ‘xriml’ macro Stata module [78] with cubic spline. Although the 95th or 97.7th percentiles [80, 81] are used as cut-offs for excluding overweight individuals (outliers), we used the conservative 1st and 99th percentiles for the exclusion of outliers to allow for more generalisability of the curve for PQ dosing in the South East Asian population. In other words, our conservative approach excluded only 2 % of individuals as outliers compared to 5 % or 10 % in other studies.

Ages ≥ 12 months were rounded down, e.g. a child aged 15 months was rounded down to 1 year; ages <12 months were expressed as a decimal of 1 year. The mg base/kg dose of PQ received for a given age group was calculated by simple division: PQ dose/all the weights for that age group.

### Data analysis and determining optimal age-dosing categories

Within each age-dosing group, we calculated the proportions of males and females, individually and combined, who would receive therapeutic doses; male-female differences were compared by the χ^2^ test. Differences in the distribution of skewed data were compared using the Mann–Whitney U test and mean differences in normally distributed data were compared using the unpaired ‘t’ test. A *P* value ≤ 0.05 was considered statistically significant.

We tested different PQ doses (1–20 mg) using different age groupings and analysed the results as above. The final age categories were selected based on (1) the mg base/kg dose received, (2) the proportions receiving a therapeutic dose, (3) how well a given age-dosing group fitted in with the next age-dosing group, (4) taking into account currently available PQ tablet strengths, even those not produced to Good Manufacturing Practice: 2.5, 5, 7.5 and 15 mg base, and (5) a desire to minimise the number of dosing groups.

When the proportions of patients receiving a therapeutic dose were similar for similar age groupings, preference was given to a supratherapeutic dose over an underdose because the risk of clinically-significant acute haemolysis was considered low, a higher dose may compensate for poor PQ metabolisers, and this strategy would allow for an increase in weight of the population in the future.

## Results

### Features of the anthropometric database

The raw database contained 28,933 individuals aged from birth onwards. After removal of missing data and data modelling, the final database included 28,138 individuals aged ≥ 6 months; the data sources are shown in Fig. 2. The proportion of females was 65.1 % (18,314/28,138); data on sex was missing for 340 individuals (1.2 %). Children aged 0.5–4, 5–12, and 13–17 years accounted for 20.0 % (n = 5640), 9.1 % (2559), and 9.1 % (2550) of individuals, respectively, while adults accounted for 61.8 % (17,389). Adults aged > 40–80 and > 50–80 years numbered 4318 (15.3 %) and 903 (3.2 %), respectively.

Excluding the 1247 patients with CNS infections whose location could not be confirmed, the urban:rural ratio was 1:4.1. All 5244 urban dwellers were healthy individuals, whilst 17,528 and 4119 rural dwellers were well and had malaria, respectively. The modelled weight-for-age scatter plots of the malaria patients, DHS-surveyed individuals, CNS infection patients, and dog bite victims overlapped substantially (Additional file 2: Figure S2). Moreover, the mean (standard deviation) weights between urban and rural dwellers were similar: 42.2 kg (19.4) vs*.* 40.7 kg (18.3), respectively, but the small mean difference of 1.5 kg (95 % CI, 0.9–2.1) was significantly different (*P* < 0.001).

### Four optimised dosing groups

We identified four optimised age dosing groups (Table 1). Overall, 90–97 % of patients would receive a therapeutic dose and the median dose received would range from 0.23 to 0.29 mg/kg of PQ base.

**Table 1:** Summary of the proportions of individuals who would receive a primaquine (PQ) dose within the defined therapeutic dosing range of 0.15–0.38 mg base/kg body weight, an underdose (<0.15 mg base/kg), and a supratherapeutic dose (>0.38 mg base/kg)

Age band, years	Weight for age			PQ dose mg base	Underdose	Therapeutic dose	Supratherapeutic dose	PQ mg base/kg		
	1st	median	99th		n (%)	n (%)	n (%)	1st	median	99th
0.5–4 (n = 5640)	6.5	11.0	17.1	2.5	89 (1.58)	5,494 (97.41)	57 (1.01)	0.15	0.23	0.38
5–9 (n = 1669)	11.0	17.0	28.8	5	5 (0.3)	1,511 (90.53)	153 (9.17)	0.18	0.29	0.45
10–14 (n = 1508)	19.0	28.0	49.0	7.5	8 (0.53)	1473 (97.68)	27 (1.79)	0.15	0.27	0.39
≥15 (n = 19,321)	36.0	51.1	74.9	15	0	18,489 (95.69)	832 (4.31)	0.20	0.29	0.42

Corresponding mg base/kg doses of PQ are shown for the 1st, 50th (median), and 99th centiles

### Optimal PQ dose breakdown by individual ages and sex

**Fig. 3:**
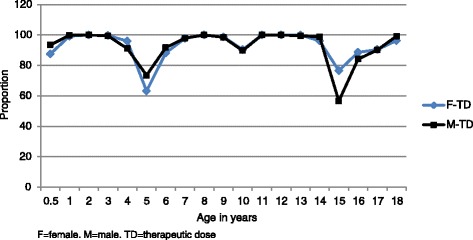
The proportions of females and males who would receive a therapeutic (0.15–0.38 mg base/kg) dose of primaquine

**Fig. 4:**
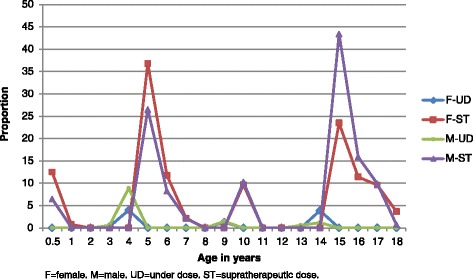
The proportions of females and males who would receive either an under (<0.15 mg base/kg) or supratherapeutic (>0.38 mg base/kg) dose of primaquine

The proportions of females who would receive a therapeutic dose ranged from 63.2 % (age 5 years) to 100 % (Fig. 3); 10 % or more of individuals aged 0.5 to < 1, 5, 6, 15, and 16 years would receive supratherapeutic doses and ≤ 4 % of individuals aged 3, 4, 9, and 14 years would be under-dosed (Fig. 4).

The proportion of males who would receive a therapeutic dose ranged from 56.6 % (15 years) to 100 % (Fig. 3); 10 % or more of individuals aged 5, 10, 15, and 16 years would receive supratherapeutic doses and ≤ 9 % aged 3, 4, 9, 13, and 14 years would be under-dosed (Fig. 4). Statistically significant results were found for male/female comparisons for ages 0.5 to < 1, 4, 5, 15, and ≥ 18 years.

**Fig. 5:**
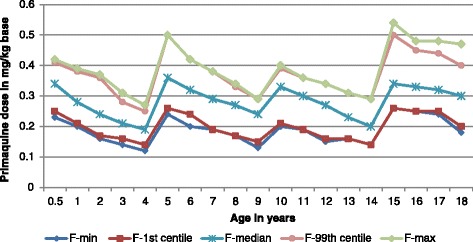
The mg base/kg dose of primaquine expressed as the lowest, highest, 1st, 99th, and 50th (median) centiles as a function of age in females

**Fig. 6:**
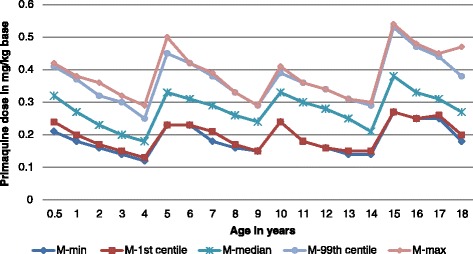
The mg base/kg dose of primaquine expressed as the lowest, highest, 1st, 99th, and 50th (median) centiles as a function of age in males

The median mg base/kg dose of PQ base ranged from 0.19 (age 4 years) to 0.36 (5 years) for females and 0.18 (4 years) to 0.38 (14 years) for males (Figs. 5 and 6). The lowest and highest mg base/kg doses were the same for females and males: 0.12 mg base/kg (4 years) and 0.54 mg base/kg (15 years).

### Weight-based PQ dosing regimen

A suggested weight-based dosing chart using weight multiples of 10 or 15 kg and the same tablet strengths as the age-based regimen was developed. Most dosing categories receive therapeutic doses (0.15–0.38 mg/kg of PQ base) except for children who weigh < 10 kg (Table 2).

**Table 2:** A suggested weight-based dosing scheme for single low-dose primaquine

Weight in kg	Dose of primaquine base in mg	Dose of primaquine base in mg/kg
5–14.99	2.5	0.5–0.17
15–29.99	5	0.33–0.17
30–39.99	7.5	0.25–0.19
40–79.99	15	0.38–0.18
80–109.9	20	0.25–0.18

## Discussion

This is the first, optimised, age-based regimen for SLDPQ and was developed using a substantial database of some 28,000 Cambodians across the age, sex, urban, and rural spectra. It has four dosing bands that could be adopted by the Centre National de Malariologie and, if suitable, adopted also by other malaria control programmes in the GMS as part of their urgent efforts to control and eliminate ARPf.

The data were obtained from several sources and included data from the two-stage, cluster-randomised nationwide DHS survey, dog bite victims (likely also to be a random sample in Cambodia), village surveys in malaria-endemic areas (some using PCR), and malaria patients. The data from patients with suspected CNS infections are likely to be the most biased samples. However, all of the assembled data overlapped substantially and the difference in weight between urban and rural dwellers was small and not significant.

Symptomatic malaria is a rural disease in Cambodia and affects mostly young adult males who work in forests. However, there are also pools of asymptomatic, submicroscopic falciparum malaria cases (detectable by PCR) affecting all ages and both sexes across many rural regions of Cambodia [82–85]. Our weight-for-age database reflected that epidemiology, containing approximately 80 % rural dwellers, malaria patients, and asymptomatic parasite-infected individuals. Thus, our dosing regimen is applicable to malaria patients and, if MDA becomes policy, asymptomatic malaria parasite carriers as well.

Although our data are unsuitable as reference data to construct a weight-for-age “growth chart” for Cambodia as a whole, we did construct “growth curves” using Box Cox modelling, which enabled us to calculate the mg base/kg dose of PQ that individuals would receive. Our weight-for-age curve is similar to those of Cambodia, Laos, and Myanmar constructed by Hayes et al. [22]. These countries are also affected by ARPf and share similar G6PDd variants with Cambodia [86]. Thus, our SLDPQ regimen is generalisable to two of Cambodia’s GMS neighbours.

There are some limitations to our study. Children aged < 5 years and women of child-bearing age predominated because most of these data came from the DHS survey. Previous databases have suffered from having fewer individuals aged 5–15 years [21] and anthropometric data from several of the databases used by Hayes et al. [22] were also somewhat patchy. Our database had relatively fewer data in the 5–15 years age group, with individual ages numbering between 200 and 300. In common with other malaria-endemic countries, individuals often do not know their exact dates of birth but are usually able to estimate their ages reasonably well. This is more important for children and help with age determination can be obtained from “Road to Health” charts and, in some countries, national ID cards.

Although we believe our SLDPQ regimen is accurate based on current knowledge of PQ’s PK and PD characteristics, new knowledge could result in its revision. Several significant knowledge gaps exist, notably, the lack of PK OM data, dose-response curves for mosquito infectivity and red cell haemolysis, and important PK parameters such as the maximum concentration or area under the concentration-time curve. There are no PQ PK data in children aged < 5 years. Yet, these data are crucial to establish whether the target PQ dose of 0.25 mg base/kg applies to this vulnerable group.

Our knowledge of PQ drug interactions is also limited. Increased awareness of drugs that could potentiate the haemolytic toxicity of SLDPQ is especially important either by a metabolic interaction at the CYP level or by direct red blood cell toxicity. Once SLDPQ is deployed, good pharmacovigilance systems will need to be in place so confidence in SLDPQ by the populace remains high. This is especially so when SLDPQ is used as MDA because healthy individuals will take a drug with the intention of a community rather than individual benefit, but which could result in toxicity for individuals. Moreover, accidental exposure to PQ in pregnancy can be expected and is another critical area of pharmacovigilance.

The effect over time of the widespread use of SLDPQ on gametocyte sensitivity and *P. vivax* liver hypnozoites is unknown. This would need to be studied to enable malaria control programmes to adapt their strategies if PQ resistance were to develop.

Currently, in Cambodia, approximately 30 % of all malaria treatments are given by village malaria workers. They will need to be trained on how to administer SLDPQ, what advice to give to recipients regarding PQ toxicity, and how to manage visibly malnourished or overweight children. As countries move towards malaria elimination and adopt MDA with SLDPQ, it is clear that the efficient dosing of large numbers of individuals is best achieved using an age-based regimen, much in the same way as anthelminthic MDA is done. Coordination between different MDA programmes would be essential.

Few manufacturers produce PQ to international Good Manufacturing Practice. Current tablet strengths are limited to 7.5 and 15 mg of PQ base, which is quite inadequate. Moreover, there are no PQ formulations for children and PQ has a very bitter taste. Options to increase palatability include sweetened dispersible tablets, as has been developed for AL [87], granules, mini tablets, and gel coating [88, 89]. These are issues of great importance that could substantially affect access to PQ and the success of malaria elimination. If regional- or country-specific, age-based dosing regimens could be designed that use a limited number of the same tablet strengths, this would be a strong argument in convincing pharmaceutical companies to engage positively in malaria elimination.

South American populations weigh more than their African counterparts, who in turn weigh more than South East Asian/Western Pacific populations [22]. Therefore, our SLDPQ regimen cannot be generalised to South America and Africa. Caution is also needed for the Middle East and parts of west Asia where anthropometric characteristics need further study and where the most severe G6PDd variant – Mediterranean – is not uncommon [33].

Our SLDPQ regimen has four age-dosing groups and we have proposed a weight-based regimen with four (five if very heavy individuals are separated off) weight bands. Neither regimen fits in neatly with commonly used ACTs. Artesunate-mefloquine, artesunate-amodiaquine (not used currently in Cambodia), and DHAPP have four, four, and six age-based dosing regimens, respectively, and AL and artesunate-pyronaridine both have four weight-based regimens. This underlies the need for good training when the age-based PQ regimen is eventually deployed [20].

## Conclusions

We have designed an age-based regimen for SLDPQ that would be suitable for use in Cambodia and other GMS countries. This regimen should be deployed on a research footing to see if it is acceptable to health workers and patients. Other SLDPQ regimens need to be developed for South America, Africa, and the Middle East/west Asia based on evidence of efficacy and tolerability. Pharmaceutical companies need to engage enthusiastically in malaria elimination.

## Additional files

Additional file 1: Figure S1. Weight-for-age modelled as a growth curve. (DOCX 46 kb)
Additional file 2: Figure S2. Scattergram of the modelled weight-for-age distributions for different groups in the database. (JPG 50 kb)

## Abbreviations

ACT: artemisinin combination therapy
AHA: acute haemolytic anaemia
AL: artemether lumefantrine
ARPf: artemisinin-resistant *Plasmodium falciparum*
CNS: central nervous system
cPQ: carboxyprimaquine
CYP: cytochrome
D: day
DHAPP: dihydroartemisinin-piperaquine
DHS: Demographic Health Survey
G6PDd: glucose-6-phosphate dehydrogenase deficiency
GMS: Greater Mekong Subregion
Hb: haemoglobin
MAO: monoamine oxidase
MDA: mass drug administration
MetHb: methaemoglobinaemia
MTB: minimum transmission blocking
OM: oxidative metabolite
PD: pharmacodynamics
PK: pharmacokinetics
PQ: primaquine
SLDPQ: single low-dose primaquine
WHO: World Health Organization
