# Erratum to: An optimised age-based dosing regimen for single low-dose primaquine for blocking malaria transmission in Cambodia

**DOI:** 10.1186/s12916-016-0765-5

**Published:** 2016-12-20

**Authors:** Rithea Leang, Naw Htee Khu, Mavuto Mukaka, Mark Debackere, Rupam Tripura, Soy Ty Kheang, Say Chy, Neeraj Kak, Philippe Buchy, Arnaud Tarantola, Didier Menard, Arantxa Roca-Felterer, Rick M. Fairhurst, Sim Kheng, Sinoun Muth, Song Ngak, Arjen M. Dondorp, Nicholas J. White, Walter Robert John Taylor

**Affiliations:** 1 National Center for Parasitology, Entomology and Malaria Control, Corner St. 92, Trapeng Svay Village, Sangkat Phnom Penh, Thmei, Khan Sen Sok, Phnom Penh, Cambodia; 2 Mahidol Oxford Tropical Medicine Research Unit (MORU), 420/6 Rajvithi Road, Rajthevee, Bangkok, 10400 Thailand; 3 Oxford Centre for Tropical Medicine and Global Health, Nuffield Department of Medicine Research Building, University of Oxford, Old Road Campus, Roosevelt Drive, Oxford, OX3 7FZ UK; 4 MSF Belgium Cambodia Malaria Program, #19, Street 388, Sangkat Tuol Svay Prey, Khan Chamkarmon, PO Box 1933, Phnom Penh, Cambodia; 5 University Research Co., LLC, MK Building, House #10 (2nd floor), St. 214, Chey Chumneas, Daun Penh, Phnom Penh, Cambodia; 6 University Research Co., LLC Washington DC: 7200 Wisconsin Ave, Bethesda, MD 20814 USA; 7 Institut Pasteur du Cambodge, 5 Monivong Boulevard, PO Box 983, Phnom Penh, 12201 Cambodia; 8 Malaria Consortium, House #91 Street 95, Boeung Trabek, Chamkar Morn, Phnom Penh, Cambodia; 9 Laboratory of Malaria and Vector Research, National Institute of Allergy and Infectious Diseases, National Institutes of Health, Rockville, MD 20852 USA; 10 FHI 360 Cambodia Office, #03, Street 330 Boeung Keng Kang III Khan Chamkamon, PO Box: 2586, Phnom Penh, Cambodia; 11 Centre de Médecine Humanitaire, Hôpitaux Universitaires de Genève, Genève, Switzerland

## Erratum

After publication of the original article [1], it came to the authors’ attention that there was an error in the **PQ pharmacokinetics** sub-section of the **Background** section. The following sentence is affected:

“There is no PK interaction between PQ and either artesunate-pyronaridine [76] or mefloquine [69, 77]; no PK interaction data exist for PQ and artemether-lumefantrine (AL).”

This sentence should have read as follows:

“AS pyronaridine increased PQ exposure by 15% without affecting significantly cPQ exposure [76]. There is no PK interaction between PQ and mefloquine [69, 77]; no PK interaction data exist for PQ and artemether-lumefantrine (AL).”
