# Supplementary figures and images for: An optimised age-based dosing regimen for single low-dose primaquine for blocking malaria transmission in Cambodia
Source: BMC Med. 2016 Oct 27;14:171. doi: 10.1186/s12916-016-0701-8 (PMC5081959; doi:10.1186/s12916-016-0701-8)

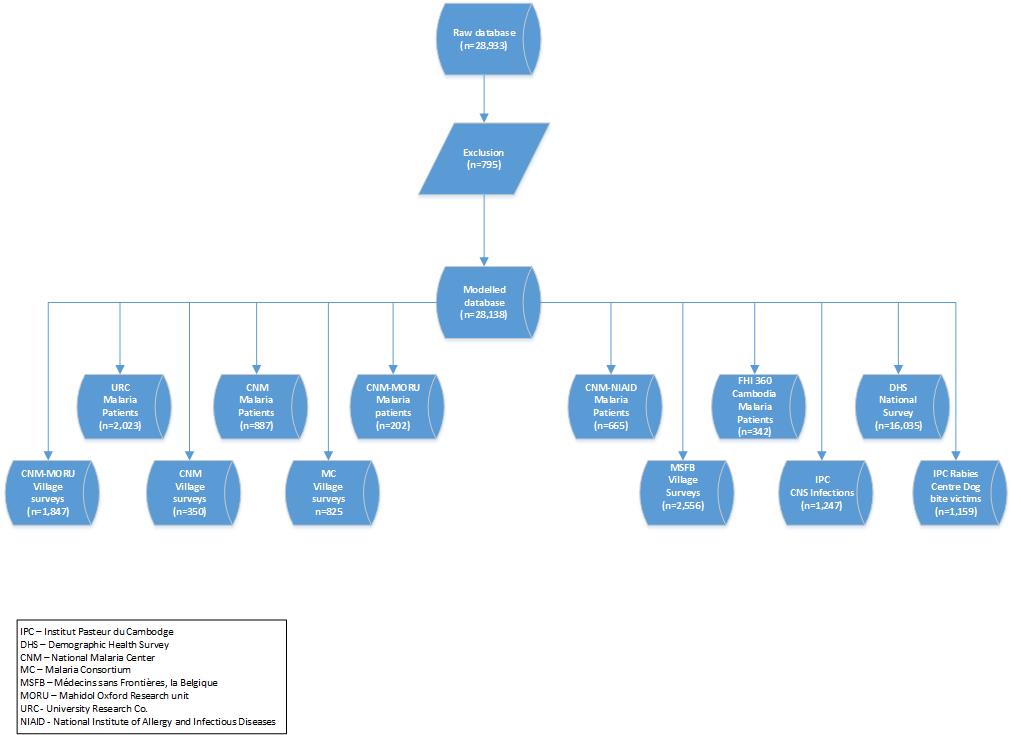

Supplement: Additional file 2: Figure S2. — Scattergram of the modelled weight-for-age distributions for different groups in the database. (JPG 50 kb) [file 12916_2016_701_MOESM2_ESM.jpg]
