# Supplementary material for: An optimised age-based dosing regimen for single low-dose primaquine for blocking malaria transmission in Cambodia
Source: BMC Med. 2016 Oct 27;14:171. doi: 10.1186/s12916-016-0701-8 (PMC5081959; doi:10.1186/s12916-016-0701-8)
Supplement: Supplementary file 2 [file 12916_2016_701_MOESM1_ESM.docx]

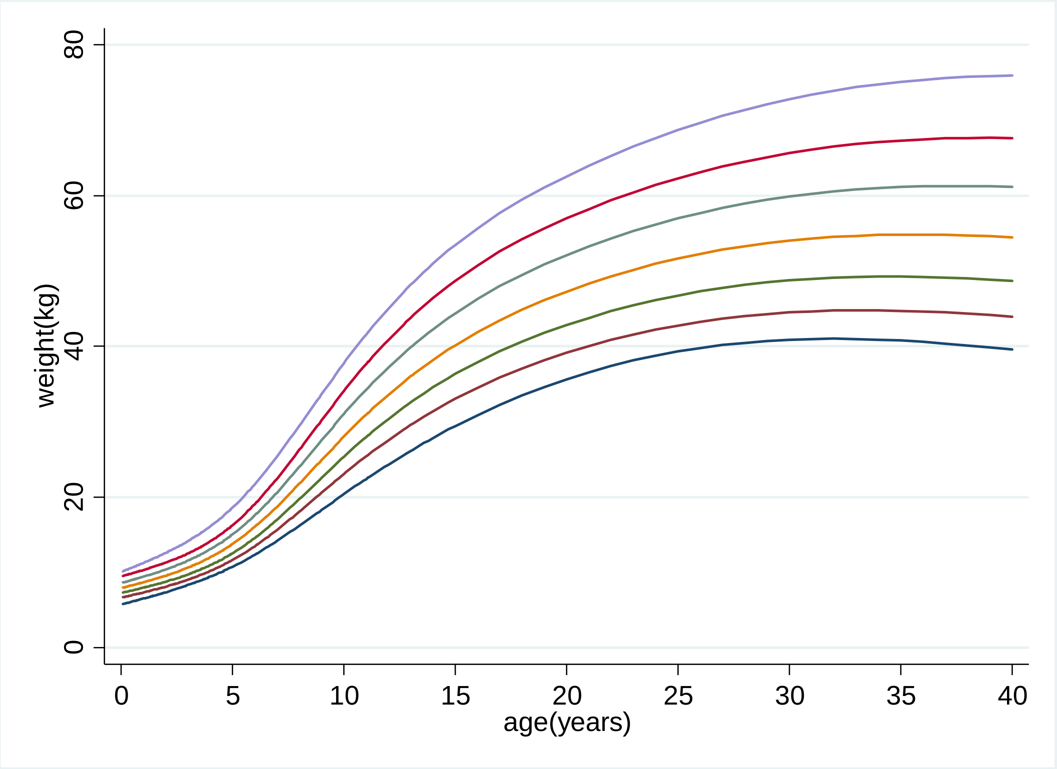

weight(kg)
0
20
40
60
80
0
5
10
15
20
25
30
35
40
age(years)
